# Supplementary figures and images for: Response of Chironomids to Key Environmental Factors: Perspective for Biomonitoring
Source: Insects. 2022 Oct 7;13(10):911. doi: 10.3390/insects13100911 (PMC9604178; doi:10.3390/insects13100911)

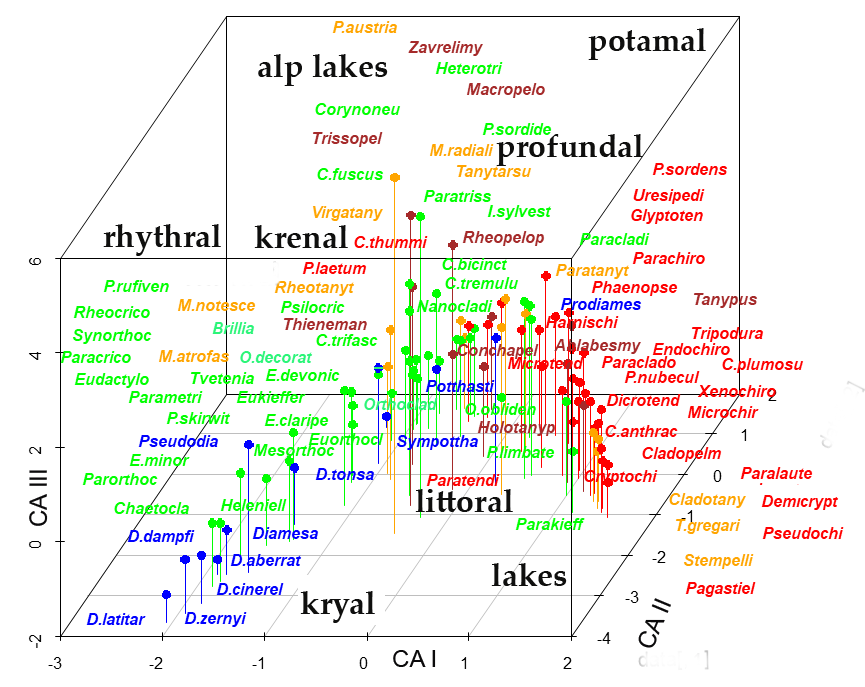

Supplement: Supplementary file 1 [file insects-13-00911-s001.zip › Fig_S1.TIF]

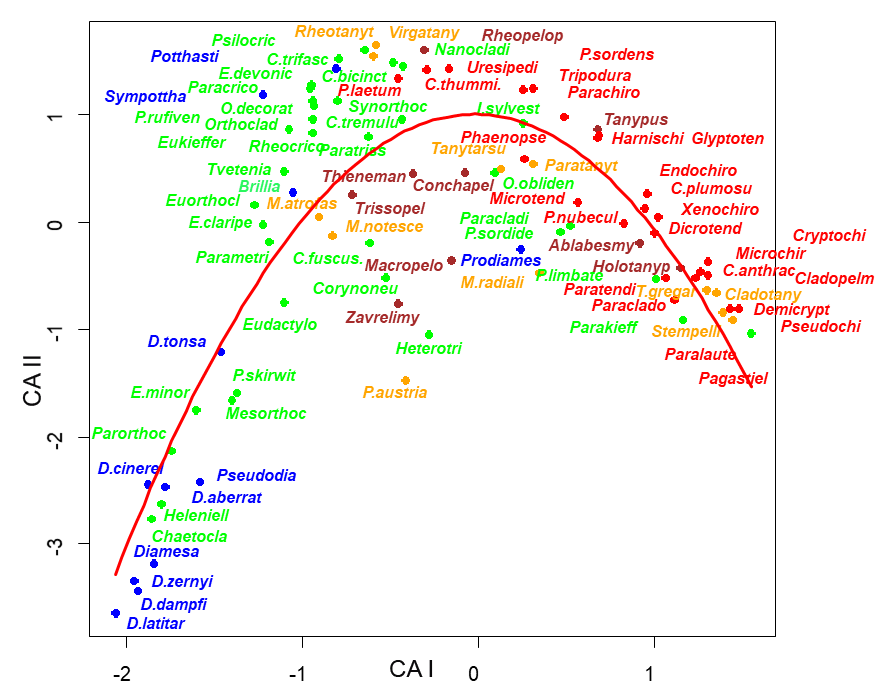

Supplement: Supplementary file 1 [file insects-13-00911-s001.zip › Fig_S2.TIF]

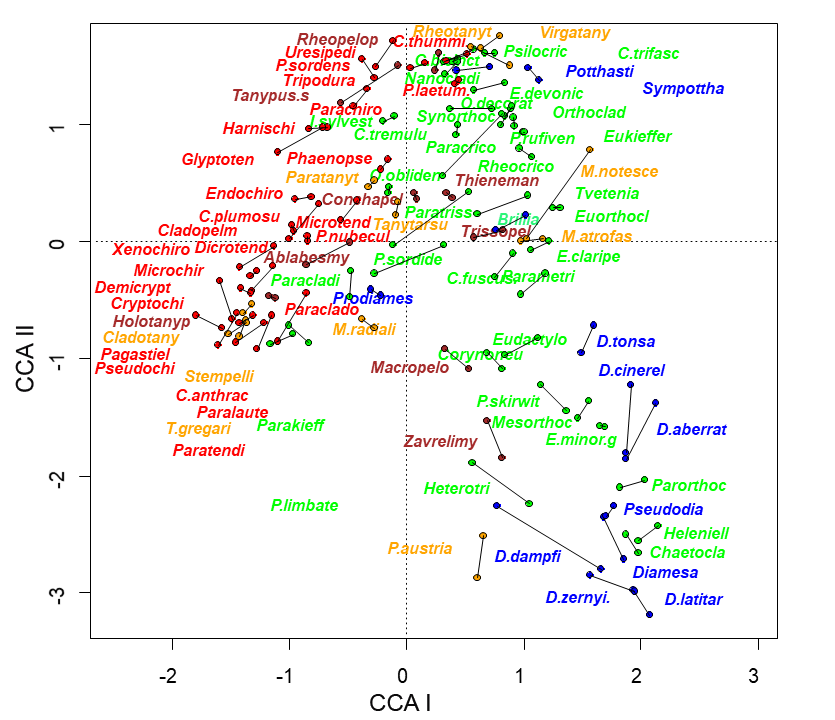

Supplement: Supplementary file 1 [file insects-13-00911-s001.zip › Fig_S3.TIF]

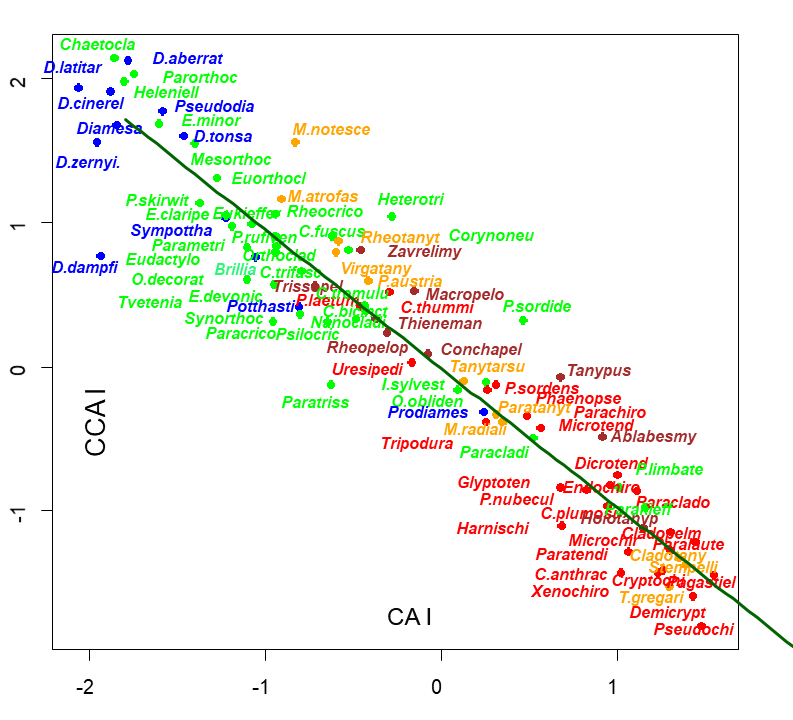

Supplement: Supplementary file 1 [file insects-13-00911-s001.zip › Fig_S4.tif]
